# Evaluation of a supportive care app for coordinating caring networks: an analysis of the first 19,000 users

**DOI:** 10.3389/fdgth.2023.1063277

**Published:** 2023-05-17

**Authors:** Ben Singh, Susan Palmer, Carol Maher

**Affiliations:** ^1^ Alliance for Research in Exercise Nutrition and Activity (ARENA), University of South Australia, Adelaide, SA, Australia; ^2^ Research and Development Department, The Gather Group Co, Gather Group, Elsternwick VIC, Australia

**Keywords:** supportive care, digital, oncology, mobile app, technology

## Abstract

**Background:**

Major illnesses such as cancer, and other traumatic life events, can lead to sudden increases in supportive care needs. This study aimed to describe engagement, acceptability and satisfaction with a supportive care networking app under real-world conditions.

**Methods:**

A total of 10,952 individuals used the app during the study period (2018–2022). The app is designed to enable “captains” to assemble a network of friends and family members to provide timely, and individually tailored, supportive care (including assistance with tasks such as taking children to school, cooking meals, grocery shopping, and transport to appointments). Engagement was determined from server data, whilst acceptability and satisfaction were captured using purposed-designed surveys.

**Results:**

Users were mostly female (76%) and aged between 30 and 49 years (61%). The most common reason for using the app was sudden illness (web: 81%; mobile: 64%). An average of 42 tasks were requested per network, with a 32% acceptance rate. Significantly more tasks were requested (web: 52.2 tasks per network; mobile: 31.7 tasks per network; *p* < 0.001) and accepted (web: 43.2%; mobile: 20.2%; *p* < 0.001) in the web app vs. the mobile app. Task requests in the web app most commonly related to food (43% of requested tasks), social (15% of requested tasks) and children (13% of requested tasks). The task acceptance rate differed by task categories (*p* < 0.001), with tasks relating to transport, medical appointments and children accepted at the highest rates (56%, 52% and 49%, respectively). Acceptability and satisfaction data suggested that the app was well received and overall, participants were satisfied with the app.

**Conclusion:**

Findings suggested that this support care networking app achieved widespread uptake for a wide variety of supportive care tasks. Future research focused on optimizing engagement with the mobile app and examining the effectiveness of the app for improving patient and hospital outcomes is warranted.

## 1. Introduction

Major illnesses such as cancer, and other traumatic life events, can lead to sudden increases in supportive care needs. Supportive care needs may include physical, emotional, social support, psychological, informational, spiritual and practical needs, both for individuals experiencing the illness or event, and those around them, such as their children and family members (1, 2). Unmet supportive care needs are common and present an issue both for the individuals directly involved and for society. Among cancer patients, up to 93% of patients' supportive care needs go unmet (3, 4). Unmet supportive care needs are associated with increased anxiety, depression, panic, social isolation, psychological distress and poorer quality of life (QoL) (3, 5–9), and lead to reduced patient satisfaction, poorer adherence to treatments, and increased longer-term health care costs and utilization (10, 11). Programs and services that improve the provision of supportive care are needed.

Family members and friends play a crucial role in providing a support network and caregiving for individuals experiencing illness and personal crises (12). In Australia, it was estimated that 2.8 million Australians provided informal care in 2020, equating to 2.2 billion hours of care, with an economic impact of $77.9 billion (13). While in the US, it was estimated approximately 41 million family caregivers provided 34 billion hours of care, with an estimated economic impact of $470 billion in 2017 (14). The right support from family and friends can play a significant supportive role, improve outcomes and protect loved ones from secondary complications (15–18), resulting in faster recovery and reduced avoidable hospital re-admissions and post-discharge complications in various health conditions (15–19).

Previous literature has identified common practical limitations associated with supportive care provided by friends and extended family, such as it being ad-hoc in nature, unstructured, uncoordinated and short-term (20–25). Technology may provide an opportunity to address these limitations, for example, by assisting recipients to identify and articulate their care needs, improving the coordination of care, improving responsiveness to the changing needs (of both the recipient and their “caring network”, e.g., due to deterioration or carers going on holidays or personal circumstances changing), building the capability of informal carer networks through curated education and information, and initiating and facilitating open lines of communication among caring networks and the recipient.

A previous systematic review (26) of digital-based supportive care interventions for people with cancer identified a range of tele-education, video-counselling, web-based, social networking approaches. Taken together, there was evidence that such support can improve QoL and functional capacity, reduce symptoms of pain, fatigue and depression (26). However, the studies identified in the systematic review were highly “research-orientated”, i.e., created for evaluation in a research trial, were evaluated in relatively modest sample sizes (over half of the studies (12/20) involved <200 participants, with the largest study involving 516 participants (27). Importantly, like many previous systematic reviews of digital self-management tools, only 15% (3 of 20) are currently available (with most either never officially released or having been discontinued) (26). E-Health experts have suggested that researchers and clinicians seek out and evaluate existing platforms, to help address these limitations (28).

The Gather Group Co. technology (https://gathergroup.com.au/; hereafter referred to as the Gather Group app) is software designed specifically for establishing and coordinating caring networks for people experiencing a health or personal crisis (e.g., cancer, stroke, surgery, accident, injury). The overall functions of the app are to assist in identifying the care needed, manage the logistics of coordinating informal support networks with the ability to respond quickly to changing needs and facilitate open lines of communication between the recipient and caring network. This study represents a collaboration between the developers of the Gather Group app and university researchers, which aims to describe the Gather Group app's first *n* = 19,104 users (Web app: *n* = 10,694; mobile app: *n* = 8,410), examine engagement with the software, and evaluate acceptability and satisfaction.

## 2. Methods

### 2.1. Study design

This is a retrospective mixed-methods study, based on data collected from users of the web and mobile app between February 2018 and September 2022. In the Terms of Use, participants provided consent for their data to be used for program evaluation purposes. As a retrospective analysis of quality assurance data, this analysis was deemed to be exempt from requiring ethics approval by the University of South Australia's Human Research Ethics Committee (application no. 204804). This manuscript is reported following STROBE guidelines, and the Gather Group Co. technology intervention is described in accordance with the TIDieR checklist.

### 2.2. Description of the Gather Group Co. technology web and mobile apps

**Figure 1:**
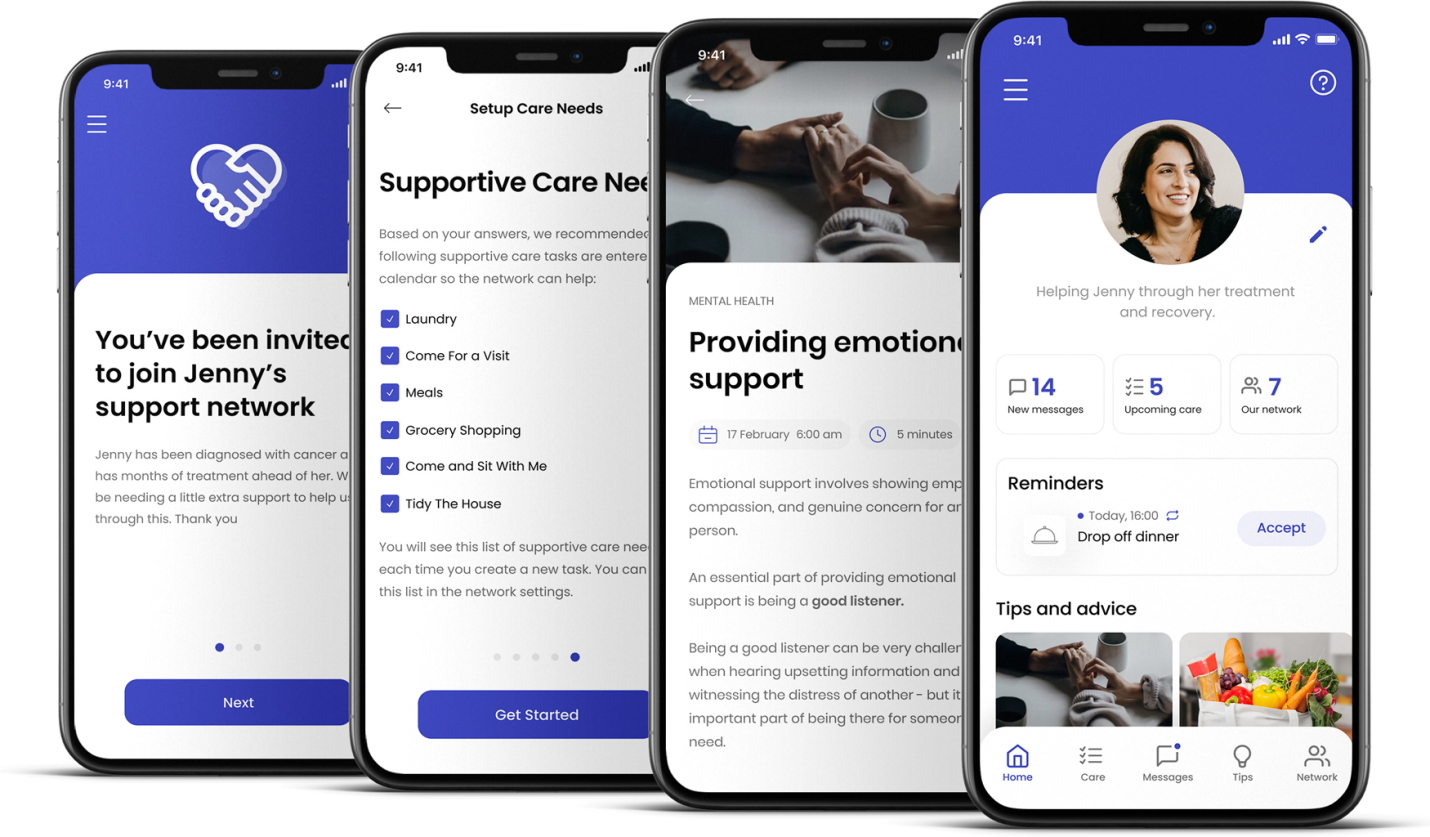
Overview of app dashboard.

The Gather Group app (Figure 1) was developed based on literature reviews and formative work overseen by a steering group of patients (cancer patients) and clinicians (social workers; psychologists; clinical nurse specialists; occupational therapists). A group of 12 cancer patients involved in the development of the app were approached through a Queensland non-profit supporting breast cancer patients. Interested patients volunteered to become part of our patient steering group who were consulted and involved in every stage of the development. From testing the concept, to developing the needs list, to testing the beta product and providing feedback on usability. The app was originally a web-app, with a beta release in early 2016, followed by a 6-month pilot with 200 cancer patients and their networks. The app was initially designed for use with cancer patients and was later expanded for the use of anyone experiencing any health or personal crisis. To be applicable to other patient groups, the evolution of the app occurred once the app was in market and based on the strong user feedback from other patient groups. We collected the feedback from specific user groups and, along with desktop research and reviewing the literature, revised the app to meet their needs, *via* our support staff and through follow-up email communication. This predominantly related to moving towards less cancer-specific language throughout the app and adding relevant unmet needs identified by users and the literature for other illnesses and issues. Changes included adaptations used in the language of the app to be more general rather than cancer specific (e.g., “cancer” replaced with “serious illness or injury”) and the “supportive care needs list” (i.e., the prompt users receive to identify the supportive care required) was expanded to include issues that relate to a wider range of health situations. The app has gone through iterative cycles of troubleshooting, updating and improvement, including being released as a smartphone app (iOS and Android OS) in 2022.

**Overview of use**: An overview of the key features of the app are shown in Table 1.

**Table 1:** Overview of the main features of the app.

Feature	Benefit
**Activation** Quick click through to join “caring network”	An activation feature maximises the supportive care connections that already exist within a community by inviting all those people who have said “let me know how I can help”.
**Need Identification & Supportive Care Plan** Customised onboarding questions identify risk factors and inform a personalized “supportive care plan” based on evidence.	The “supportive care plan” highlights the specific needs of the patient and identifies the supportive care tasks that will have the most impact on their coping, their physical health and emotional health. This feature also triggers family members and friends to think more broadly about the supportive care that can be provided, with a strong focus on social connection and emotional health.
**Support Coordination** Simple “roster” that lists the help needed to be accepted by “carer network”	A transparent supportive care roster enables care networks to choose what they can do around their own commitments and at a time that suits them—increasing the likelihood of ongoing supportive care. It provides the patient with certainty that ongoing and regular support is available—reducing anxiety and building a greater sense of safety and connection. The transparent nature of a roster system allows the carer network to see the level care and help that is needed and can motivate change and additional support.
**Enhanced Communication** Shared communication channel that is controlled by the administrator and managed within folders	The communication channel allows members of the carer network to communicate about issues and updates relating to treatment, prognosis, care and support. This shared communication platform serves to (i) reduce misinformation or situations when information is lost altogether/not shared among the group; (ii) make it easier for the patient or “carer network” to initiate a topic that they may find challenging to do in person; (iii) keep information filed in “folders” for easy access
**Education & Information** Targeted education and support to improve the quality of care and the confidence of the caring network.	The Education and Information feature provides updated information and guidance to the caring network from social workers, psychologists and clinical nurse specialists *via* the customer dashboard. It is focused on improving the supportive care available to patients by changing the behaviours of friends and family through better education and awareness of issues and needs. Enhancing the knowledge base of the personal network allows them to be informed and support the patient with decision-making and life changes.

**Captain**: The app is designed to be used by a captain (i.e., the individual who is experiencing the health or personal crisis, or a friend or family member of an individual experiencing a health or personal crisis) who coordinates the care network.

**Care network**: friends, family or community members. Captains first download the app from the Apple App/Google Play stores then create a care network by inviting friends, family members or community members to join their care networks via a step-by-step process in the app. In the mobile app, the number of people who could join a network was restricted to a maximum of 10. This was done in response to an observation that, in some instances, very large networks were formed on the web app (e.g., over 100 members), and that these very large networks did not appear any more effective than smaller teams.

**Identification of supportive care needs**: The captain completes a series of questions to help identify the supportive care needs (What brings you here? Who needs help? Which of the following apply to the person needing help? What tasks do they need assistance with? (e.g., take kids to school; come for a visit; family meals; grocery shopping; transport to appointments; help with laundry)). This includes pre-emptively identifying areas of unmet need that are linked to a poorer outcome (e.g., depression, anxiety, stress, distress, coping, treatment adherence and family breakdown) and creating a list of supportive care needs that are recommended to the captain.

**Task rostering/Calendar**: The supportive care needs are then turned into a roster of practical tasks by the captain. These tasks, including days/times and additional information, are visible to the support network via their personal account and can be accepted by the network based on their own availability. Descriptions for each task can be added by the captain, to provide the network members with additional information about the task.

**Communication feature**: The app includes a communication feature, with a real-time chat function, and where users can upload relevant documents or files such as past weekly shopping lists, recipes, news and photos, and brainstorm new ways to provide help.

**Figure 2:**
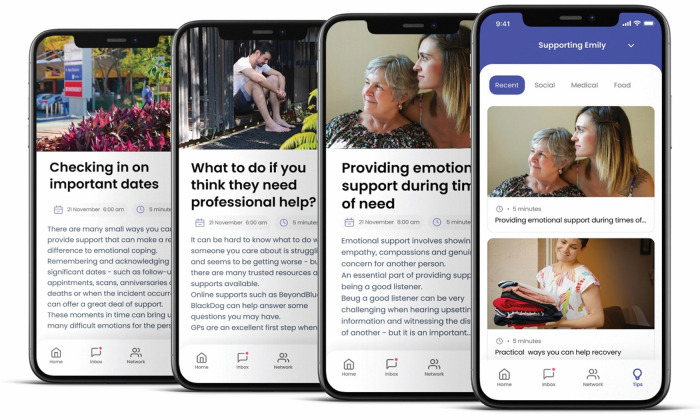
Tips sent by clinical experts.

**Education feature**: Clinical experts (social workers; psychologists; clinical nurse specialists) send the care network tips on how they can best provide care based on evidence-based practice (Figure 2), designed to improve care and patient outcomes. The tips are designed to address specific needs and changing circumstances.

**Notifications**: Users receive notifications for unallocated tasks, where they have the option to accept the tasks. If a user accepts a task, they then receive notifications for upcoming tasks as a reminder to complete the tasks.

### 2.3. Setting

The initial user testing of the app was conducted at three Australian hospitals in the major cities: Melbourne, Brisbane, and Sydney. Development of the app was undertaken by a Melbourne-based IT company. Release of the app involved users Australia-wide and assessment of app users was conducted between February 2018 and June 2022.

### 2.4. Participants (Eligibility and recruitment)

Breast cancer patients and oncologists at three Australian hospitals (in Melbourne, Brisbane, and Sydney) were invited to participate in assessing the feasibility of the concept for the app, and initial user testing. The app was then publicly released, and available for download and use by anyone in need of support. Users were adults aged 18 years or older, who were either directly experiencing a health or personal crisis (e.g., cancer, stroke, surgery, accident, injury) requiring supportive care, or were a friend or family member of a person who was experiencing a health or personal crisis requiring supportive care. For the web app, users needed access to a computer or mobile device with internet access. For the mobile app (2022 onwards), users needed an iOS or Android OS smartphone with internet access. In addition to being freely publicly available, clinicians from three Australian hospitals informed potential patients about the app and encouraged them to download and use the app if they were in need of support.

### 2.5. Variables and data sources

Evaluation of the web and mobile apps included the assessment of (1) reach (to assess the demographics of users); (2) engagement (to assess the usability, functionalities, and features); and (3) acceptability and satisfaction.

**Reach**: The number of users was obtained from the server logs for both web app and mobile app users. When logging into the web app for the first time, users were asked to provide demographic details and medical conditions/reasons for using the app. On the mobile app, in order to reduce sign-up friction, demographic data was skippable for captains and was not asked of network members. Demographic data included age (in age group categories), gender, and postcode.

**Engagement**: To reduce the potential for recall and social desirability bias, engagement data were obtained by downloading usage data of the apps including the number of total users, and networks that were created, and the total number of tasks created and accepted.

**Acceptability, satisfaction and usability**: Acceptability, satisfaction and usability data were obtained using self-report surveys that were sent to subsamples of participants after using the app.

### 2.6. Bias

The app was promoted widely to encourage as many users as possible. Given that data were collected in an ecological (i.e., real-world) setting, no attempts were made to address bias (e.g., no attempts to balance the gender or socioeconomic status of users). Thus, the data represent real-world app users and app usage.

### 2.7. Statistical methods

Data for this evaluation were reported descriptively, using means (standard deviations) or numbers of users (and percentages). The web app was initially released, then later updated to a mobile app only. Therefore, results are presented for available data for each platform. Chi square tests were used to examine differences in count data (e.g., age group categories and gender of web and mobile app users). An alpha of 0.05 was used to denote statistical significance.

## 3. Results

### 3.1. Reach: demographics of users

A total of 19,104 individual users registered to use the app. Demographic data were available for 10,952 of them (Table 2), comprising 10,694 web app users (661 captains, 10,033 networks) and 258 mobile app users (captains only). The majority of users were female (76%) and aged between 30 and 49 years (61%). Amongst the web app users, team captains were slightly younger than network members (Chi^2^ = 11.34, df = 5, *p* = 0.045), but did not differ on the basis of sex. Comparing captains in the web app vs. captains in the mobile app, web app captains were again relatively younger (Chi^2 ^= 94.2, df = 5, *p* < 0.001) and more likely to be female (Chi^2^ = 16.6, df = 2, *p* < 0.001).

**Table 2:** Sample demographics.

Demographics	Web app Mean (SD) or *n* (%)			Mobile app January to September Mean (SD) or *n* (%)		
	Captain	Network	Total	Captain	Network	Total
**Age**						
<20	3 (0.5%)	76 (0.8%)	79 (0.7%)	8 (0.7%)	–	–
20–29	34 (5.1%)	516 (5.1%)	550 (5.1%)	13 (1.2%)	–	–
30–39	176 (26.6%)	2,445 (24.4%)	2,621 (24.5%)	40 (3.7%)	–	–
40–49	254 (38.4%)	3,652 (36.4%)	3,906 (36.5%)	77 (7.1%)	–	–
50–59	87 (13.2%)	1,541 (15.4%)	1,628 (15.2%)	54 (5.0%)	–	–
≥60	35 (5.3%)	824 (8.2%)	859 (8.0%)	66 (6.1%)	–	–
Unknown	72 (10.9%)	979 (9.8%)	1,051 (9.8%)	832 (76.3%)	–	–
**Gender**						
Female	521 (78.8%)	7,818 (77.9%)	8,339 (78.0%)	145 (13.3%)	–	–
Male	68 (10.3%)	1,236 (12.3%)	1,304 (12.2%)	30 (2.7%)	–	–
Other or rather not say	0 (0.0%)	0 (0.0%)	0 (0.0%)	3 (0.3%)	–	–
Unknown	72 (10.9%)	979 (9.8%)	1,051 (9.8%)	912 (83.7%)	–	–
SEIFA	1,036.4 ± 53.1	–	–	–	–	–
**Who is the assistance for?**						
Family member	–	–	–	–	–	183 (32.4%)
Friend	–	–	–	–	–	166 (29.4%)
Community member	–	–	–	–	–	33 (5.9%)
Yourself	–	–	–	–	–	182 (32.3%)

Reasons for use were illness (web: *n* = 8,619, 80.6%; mobile: *n* = 164, 63.5%), sudden death (web: *n* = 620, 5.8%; mobile: *n* = 15, 5.7%), new baby (web: *n* = 310, 2.9%; mobile: *n* = 11, 4.4%), natural disaster (web: *n* = 0, 0.0%; mobile: *n* = 1, 0.8%), COVID (web: *n* = 0, 0.0%; mobile: *n* = 9, 3.3%), accident (web: *n* = 310, 2.9%; mobile: *n* = 11, 4.1%), and “other” (web: *n* = 834, 7.8%; mobile: *n* = 47, 18.2%).

The mobile app collected data on who the team was assembled to help, with an even distribution of just under one third set up to support a family member (*n* = 183, 32.4%), a friend (*n* = 166, 29.4%) or the captain themselves (*n* = 182, 32.3%). The mobile app also collected data on the supportive care needs/living conditions of the care recipient (*n* = 914), with the most common care needs (risk factors) related to having frequent medical appointments (*n* = 320, 35%), dependent children (*n* = 278, 30.4%), living alone (*n* = 162, 17.7%) and owning a pet (*n* = 154, 16.8%).

### 3.2. Engagement: usage data

Usage data is shown in Table 3. Usage data were available for 1,751 unique networks (web: *n* = 661 [722 networks were created, of which 61 networks did not create tasks]; mobile: *n* = 1,090) and 19,104 network members (web: *n* = 10,694; mobile: *n* = 8,410). Networks formed within the web app were significantly larger than networks formed within the mobile app (mean of 16 vs. 8, respectively; Chi^2^ = 214.9, df = 1, *p* < 0.001). Approximately one third (34%) of web app users self-reported that they used the app for between 1 and 4 months, another third used it for longer (38%) and the other third used it for less (28%) (length of use data was not available for the mobile app).

**Table 3:** Usage data of the web and mobile app.

	Web app Mean (SD) or *n* (%)	Mobile app Mean (SD) or *n* (%)
	Total	Total
Number of networks	661	1,090
Number of network members	10,694	8,410
Average network size	16	8
**Task Data**		
Total number of tasks created	34,493	34,525
Total number of tasks accepted	14,896	7,004
**All requested tasks (breakdown by category)**		
Children	4,472 (13.0%)	–
Food	14,664 (42.5%)	–
Home	3,442 (10.0%)	–
Medical	1,234 (3.6%)	–
Pets	2,013 (5.8%)	–
Social	5,122 (14.8%)	–
Transport	1,777 (5.2%)	–
Other	1,769 (5.1%)	–
Total	34,493 (100.0%)	–
**Accepted tasks (breakdown by category)**		
Children	2,177 (15%)	–
Food	6,937 (47%)	–
Home	1,470 (10%)	–
Medical	642 (4%)	–
Pets	859 (6%)	–
Social	1,385 (9%)	–
Transport	1,003 (7%)	–
Other	423 (3%)	–
Total	14,896 (100%)	–
**Task acceptance rates**		
Children	48.7%	–
Food	47.3%	–
Home	42.7%	–
Medical	52.0%	–
Pets	42.7%	–
Social	27.0%	–
Transport	56.4%	–
Other	23.9%	–
Overall	43.2%	–
**How long was the support network active?**		
Less than a month	26 (27.66%)	–
Between 1 and 4 months	32 (34.04%)	–
Between 4 and 8 months	20 (21.28%)	–
Longer than 8 months	16 (17.02%)	–
Total responses	94 (100%)	–

On average, 42 tasks were requested per network, though the number of requested tasks per network was significantly greater for the web app than the mobile app (52.2 tasks per network vs. 31.7; Chi^2^ = 102.1, df = 1, *p* < 0.001). In addition, the web app had a higher task acceptance rate (43.2% of tasks accepted vs. 20.2%, Chi^2^ = 2180.8, df = 1, *p* < 0.001).

**Figure 3:**
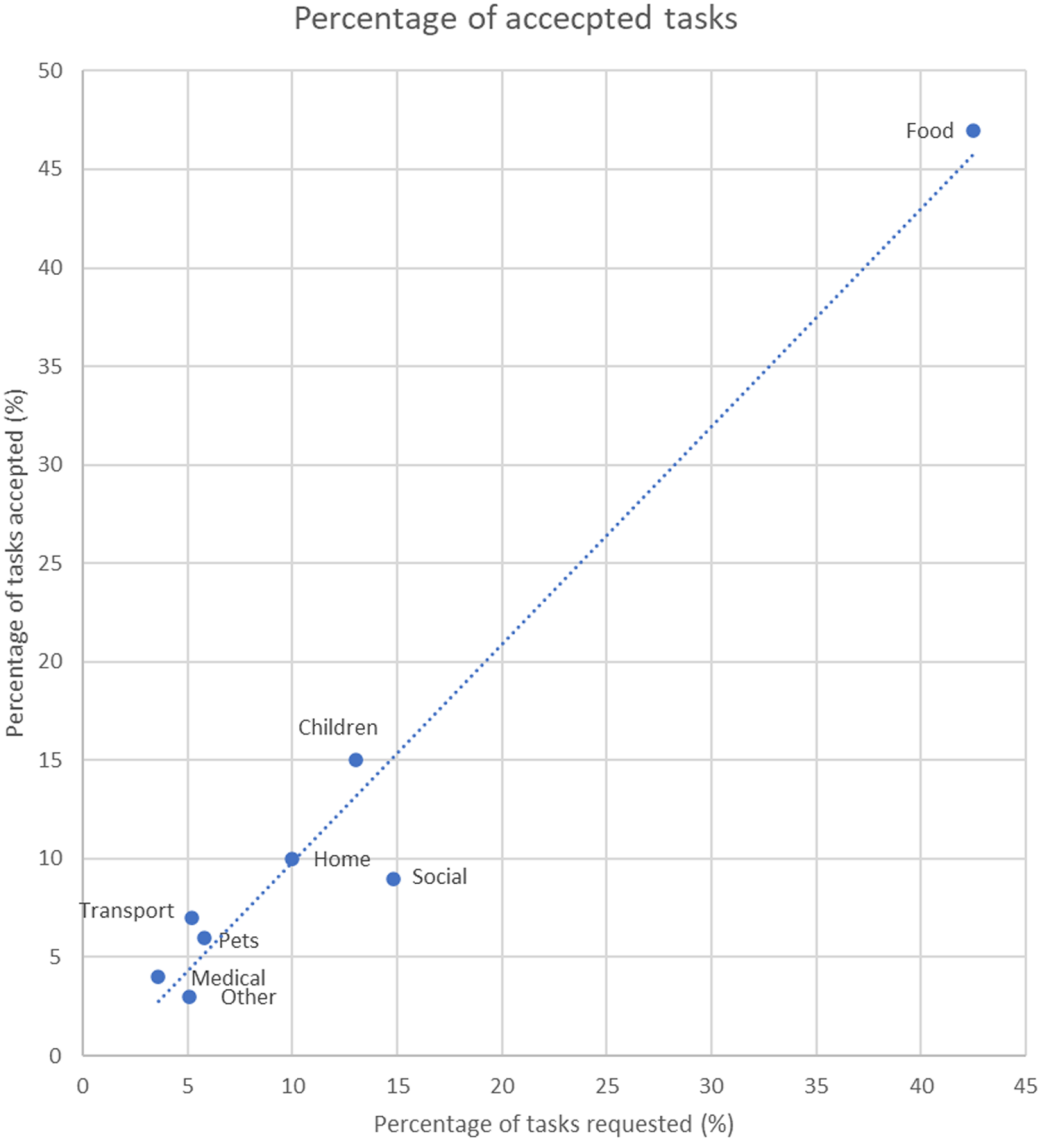
Shows the % of tasks requested and accepted for different task categories. By far, the most commonly requested and accepted task related to food (i.e., meals and grocery shopping). The diagonal line indicates agreement between the % of tasks requested and accepted in each category. Tasks above the line were most likely to be accepted, while tasks below the line were least likely to be accepted. Tasks related to food, children and transport were the most likely to be accepted, whereas tasks that were social in nature were least likely to be accepted.

The web app collected the care tasks in categories (Table 3) (whereas the mobile app did not categorize the tasks, and instead allowed them to be fully personalized). Task requests most commonly related to food (43% of requested tasks), social (15% of requested tasks) and children (13% of requested tasks). The task acceptance rate differed by task categories (Chi^2^ = 487.5, df = 7, *p* < 0.001), with tasks relating to transport, medical appointments and children accepted at the highest rates (56%, 52% and 49%, respectively), and tasks related to “other” and social accepted at the lowest rates (24% and 27%, respectively). The proportions of tasks created and accepted for the web app (broken down by category) is shown in Figure 3.

### 3.3. Acceptability, satisfaction and usability

Acceptability and satisfaction surveys were randomly sent via email to subgroups of *n* = 54 web app, and *n* = 40 mobile app active users to guide software iterations. These subsamples included *n* = 13 web app captains (24% response rate), *n* = 25 mobile app captains (63% response rate), whose results are shown in Table 4, items 1–7. In addition, a subsample of *n* = 18 users (45% response rate) who registered on the mobile app, but did not go on to use it, were surveyed about reasons why they had opted not to use the app (Table 4, items 8–15). The survey (delivered using Survey Monkey) was open for one week, and participants did not receive a reminder prompt. Web and mobile app users generally agreed or strongly agreed that using the app made coordinating help easier (web: 69.2%; mobile: 88.0%); increased the number of regular helpers (web: 61.5%; mobile: 60.0%); helped to provide the right type of help (web: 76.9%; mobile: 68.0%); helped them feel emotionally supported (web: 84.6%; mobile: 76.0%); helped to reduce the practical burden of the crisis (web: 76.9%; mobile: 84.0%); and improved communication between helpers (web: 61.5%; mobile: 84.0%). Around two-thirds of respondents said they would recommend the app (web: 69.2%; mobile: 64.0%). Of those who initially registered but did not end up using the mobile app, approximately half (53.0%) agreed or strongly agreed that it was easy to register a network on the app. The most common reasons for not going ahead with using the app were because they were “just exploring the app” (44.4%), that they had found other ways to coordinate help (33.3%) or that they felt awkward asking for help (22.2%). However, most non-users agreed or strongly agreed that they still may use the app in the future (77.8%) and that they would recommend the app to others (72.2%).

**Table 4:** Captain acceptability and satisfaction results (responses from acceptability survey) (items 1–7) and reasons why people did not end up using the app (items 8–15).

	Strongly disagree	Disagree	Neither agree nor disagree	Agree	Strongly agree	Weighted average
1. Using the app made coordinating help easier						
Web app, *n* = 13	1 (7.69%)	0 (0.00%)	3 (23.08%)	2 (15.38%)	7 (53.85%)	4.08
Mobile app, *n* = 25	0 (0.00%)	1 (4.00%)	2 (8.00%)	8 (32.00%)	14 (56.00%)	4.40
2. Using the app increased the number of regular helpers						
Web app, *n* = 13	1 (7.69%)	1 (7.69%)	3 (23.08%)	3 (23.08%)	5 (38.46%)	3.77
Mobile app, *n* = 25	0 (0.00%)	1 (4.00%)	9 (36.00%)	8 (32.00%)	7 (28.00%)	3.84
3. Using the app has helped us provide the right type of help						
Web app, *n* = 13	1 (7.69%)	0 (0.00%)	2 (15.38%)	5 (38.46%)	5 (38.46%)	4.00
Mobile app, *n* = 25	0 (0.00%)	1 (4.00%)	7 (28.00%)	8 (32.00%)	9 (36.00%)	4.00
4. Using the app has helped the person in need feel emotionally supported						
Web app, *n* = 13	0 (0.00%)	0 (0.00%)	2 (15.38%)	4 (30.77%)	7 (53.85%)	4.38
Mobile app, *n* = 25	0 (0.00%)	1 (4.00%)	5 (20.00%)	13 (52.00%)	6 (24.00%)	3.96
5. Using the app helped to reduce the practical burden of the crisis						
Web app, *n* = 13	1 (7.69%)	1 (7.69%)	1 (7.69%)	5 (38.46%)	5 (38.46%)	3.92
Mobile app, *n* = 25	1 (4.00%)	0 (0.00%)	3 (12.00%)	7 (28.00%)	14 (56.00%)	4.32
6. Using the app has improved communication between helpers						
Web app, *n* = 13	0 (0.00%)	2 (15.38%)	3 (23.08%)	4 (30.77%)	4 (30.77%)	3.77
Mobile app, *n* = 25	0 (0.00%)	0 (0.00%)	4 (16.00%)	9 (36.00%)	12 (48.00%)	4.32
7. I would recommend the app						
Web app, *n* = 13	0 (0.00%)	2 (15.38%)	2 (15.38%)	3 (23.08%)	6 (46.15%)	4.00
Mobile app, *n* = 25	1 (4.00%)	2 (8.00%)	6 (24.00%)	11 (44.00%)	5 (20.00%)	3.68
Survey of those who did not end up using the app						
8. It was easy to register a network on the app, *n* = 17	3 (17.65%)	1 (5.88%)	4 (23.53%)	5 (29.41%)	4 (23.53%)	3.35
9. I did not complete set up (invite people) because it still felt awkward to ask for help, *n* = 18	4 (22.22%)	2 (11.11%)	8 (44.44%)	4 (22.22%)	0 (0.00%)	2.67
10. I did not complete set up (invite people) because I was just checking it out, *n* = 18	5 (27.78%)	1 (5.56%)	4 (22.22%)	4 (22.22%)	4 (22.22%)	3.06
11. I did not complete set up because the app seemed difficult to use, *n* = 18	3 (16.67%)	5 (27.78%)	7 (38.89%)	1 (5.56%)	2 (11.11%)	2.67
12. I did not complete set up because I didn't want to answer the ‘help list’ questions at the start, *n* = 17	5 (29.41%)	4 (23.53%)	7 (41.18%)	1 (5.88%)	0 (0.00%)	2.24
13. I did not complete set up because I found a different way to coordinate help, *n* = 18	3 (16.67%)	3 (16.67%)	6 (33.33%)	5 (27.78%)	1 (5.56%)	2.89
14. I may still use the app in the future, *n* = 18	0 (0.00%)	1 (5.56%)	3 (16.67%)	6 (33.33%)	8 (44.44%)	4.17
15. I would still recommend the app, *n* = 18	0 (0.00%)	0 (0.00%)	5 (27.78%)	4 (22.22%)	9 (50.00%)	4.22

Feedback on the web app usability was obtained from a subsample of participants *via* an automated request when a user closed their account (*n* = 94, 19% response rate; Table 5). Results showed that two thirds of respondents were either satisfied or extremely satisfied with the reliability of the app (64.5%) and with the security of the technology (62.6%). Half of participants were satisfied or extremely satisfied with the look and feel of the app (50%).

**Table 5:** Satisfaction results (*n* = 94).

	Not at all satisfied	Not so satisfied	Somewhat satisfied	Very satisfied	Extremely satisfied	Total responses
How satisfied are you with the reliability of the app?	6 (6.45%)	8 (8.60%)	19 (20.43%)	36 (38.71%)	24 (25.81%)	93 (100%)
How satisfied are you with the security of our technology?	4 (4.40%)	2 (2.20%)	28 (30.77%)	39 (42.86%)	18 (19.78%)	91 (100%)
How satisfied are you with the look and feel of the app?	8 (8.51%)	12 (12.77%)	27 (28.72%)	31 (32.98%)	16 (17.02%)	94 (100%)

## 4. Discussion

This study represents a collaboration between commercial app developers and university researchers to understand the reach, usage, acceptability, satisfaction and usability of a supportive care app for people going through health issues and other life events. Results showed that the app users were overwhelmingly female, with the most common reasons for use being sudden illness. To date, the app has been used by 19,104 users in 1,751 networks (mean network size = 12). The Gather Group app has been used to arrange support for tasks across a wide variety of domains, most commonly assistance with food (accounting for 42.5% of requests), followed by social and children. Network members' task uptake varied by task domain, with tasks related to transport, medical appointments and children accepted at the highest rates. Some differences in the app's usage emerged between the web app and mobile app. In particular, networks assembled using the web app were larger, requested more tasks, and had a higher task acceptance rate. Acceptability and satisfaction data suggested that the app was well received and generally considered useful.

To our knowledge, and based on a search of the literature, the Gather Group app is quite unique in its features and approach. Most digital supportive care programs tend to focus on either facilitating communication between a patient and health professionals (e.g., provision of telehealth (29), remote nursing support (30, 31), counselling (32, 33) or they focus on assisting the individual to more successfully self-manage their condition (34, 35). In contrast, the Gather Group app seeks to enhance the network of family members and friends surrounding a patient to enable them to provide support. Thus, it appears that the Gather Group app addresses a gap in existing digital health supportive care tools. The large number of networks formed, taken together with the positive acceptability and satisfaction results found in this study, suggest there is considerable interest in this digital supportive care network approach.

The Gather Group app has been used to request support for a wide variety of supportive care tasks, most commonly related to food, children and home. Our results showed that particular types of tasks are significantly more likely to be accepted, while others are less likely to be accepted—namely, the tasks with the highest uptake rate related to transport, medical appointments and children, whilst those with the lowest related to social tasks or other. These findings are congruent with previous research which highlighted that health crises commonly lead to unmet life demands across a range of domains (36–38). People's requested tasks did not necessarily reflect the types of tasks that their friends or family members wanted to assist with. This may reflect the perceived importance of the tasks, or perhaps the convenience of the tasks, for example, collecting children or doing food shopping or preparing food at the same time as doing the tasks for oneself. During a personal health crisis, friends and family members often provide support for household chores, activities of daily living, and provide emotional support/companionship for the individual in need (39). It is also important to note that most participants in our study were female (76%) and previous work has highlighted gender imbalances in caregiving and unpaid work (40). Prior work indicates that a higher burden placed on caregivers can result in reductions in wellbeing of the caregiver, which may, in turn, negatively affect the ability of the caregiver to continue to assist the individual in need (41–43). Therefore, the features on the app which allowed individuals to accept or decline task requests may help ensure that an excessive burden is not placed on friends and family. Analyses highlighted some apparent differences in usage of the web app vs. the mobile app. The app has undergone many software iterations aimed at improving the app and adding new features identified as important by the steering group and based on user feedback. The most significant of these was switching from a web app platform to a mobile app platform. Results suggested that peoples' usage of the apps was somewhat different from the web vs. mobile platforms. In particular, the networks assembled were half the size, fewer tasks were requested, and the task acceptance rate was 23% lower for the mobile app compared with the web app. The finding that networks were smaller in size on the mobile app is unsurprising given that networks were restricted to a maximum of 10 members in the mobile app. As described in the methods, this was done in response to an observation that, in some instances, extremely large networks were formed on the web app, and these large networks did not appear any more effective than smaller teams. Therefore, the steering group suggested limiting the network size in the mobile app, on the rationale that a smaller network would enhance the sense of personal responsibility (44, 45), and help ensure that all team members actively contributed. It is therefore somewhat surprising that the task acceptance rate was lower for the mobile app than for the web app. One possible explanation may be that people may use web apps differently from mobile apps. Previous reports have highlighted ongoing engagement as a key challenge for mobile apps, with approximately one quarter of mobile apps only being used once, more than half of users abandoning use within 2 months (46–50). It is also prudent to acknowledge that the mobile app had only been in use for 9 months at the time of data collation for this study and during this time, a number of usability issues were identified and addressed. It is possible that this negatively impacted usage during the period studied here. Furthermore, changes in external circumstances may have influenced usage since the release of the mobile app. In particular, there have been major flooding events and community transmission of COVID in Australia in 2022. These life events may entail fewer and shorter-term supportive care needs, which may explain the lower number of tasks requested and accepted in the mobile app.

### 4.1. Strengths and limitations

A key strength of the current study is that it is a collaboration between industry and academia. Given that many academic-initiated programs fail to gain traction and are not sustained beyond their research lifespan, working with industry is an important way to achieve sustainability and real-world impact. In addition, this study examined a large sample of users, whose data reflected “real-world” usage, enhancing the ecological validity of findings. Limitations must also be acknowledged. As a commercial app with a focus on user experience, useful data (such as user demographics) was optional not mandatory, leading to large amounts of missing data. Similarly, data on the impact, usability and satisfaction with the app were collected from small subsets of users, based on self-reported recall items which emphasized low participant burden. These questions were also multiple choice, which prevented us from obtaining more detailed information on why participants were dissatisfied with the app. Furthermore, as an industry-led software product, only cancer patient end-users were involved in the app's initial design and subsequent adaptations were made in a consultative manner. Academic-led software development emphasises the use of intensive co-design in order to achieve effective, user-friendly, and patient-centred apps.

### 4.2. Future directions

In future, a more rigorous experimental study design, involving repeated measures, and a comprehensive battery of assessment tools, would provide insight into the effectiveness of the Gather Group app in enhancing supportive care. Ideally, such a trial would capture data on the impacts on the person receiving the care, their support network, and its impacts on health system outcomes.

There is also the opportunity to delve more deeply into the evolving user base of the app. The Gather Group app was initially released as a cancer supportive care program however, it is now being used for a very wide range of life events. As the user base continues to grow, it will be useful to explore the specific needs of these different user groups and explore how usage may differ between these groups.

Finally, our findings have implications for supportive care software development. Further work is required to confirm, and understand the reasons underpinning, any differences in engagement between mobile apps vs. web apps. Mobile apps offer some key functionality advantages over web apps (especially, the ability to send notifications and location-based features). At this stage, only the mobile app will be available going forward. This is because the vast majority of users express their preference for the mobile app, over the web app, which may possible be due to ease of access, ability to invite people, receiving reminders, and calendar updates. The developers are focused on responding to user feedback to improve the mobile experience. App developers must continually strive to harness the unique capabilities of mobile apps whilst also optimizing app engagement.

## 5. Conclusion

Major illnesses such as cancer, and other traumatic life events, can lead to sudden increases in supportive care needs. Family members and friends play a crucial role in providing a support network and caregiving for individuals experiencing illness and personal crises. Findings from this study showed that an app designed to establish and coordinate support networks was used to arrange support for tasks across a wide variety of domains, most commonly tasks related to assistance with food, social support and children. Acceptability and satisfaction data suggested that the app was well received and generally considered useful. Future research into optimizing engagement with supportive care mobile apps, and into the effectiveness for improving patient and hospital outcomes is required.

## Data Availability

The datasets presented in this article are not readily available because they contain protected personal and health information. Requests to access the datasets should be directed to susan-palmer@gathergroup.com.au.
